# Assessing the impact of digital health literacy on health management practices in Arab Middle Eastern and North African countries: *insights from predictive modeling*

**DOI:** 10.3389/fdgth.2025.1555436

**Published:** 2025-10-03

**Authors:** Radwan Qasrawi, Haleama Al Sabbah, Ghada Issa, Suliman Thwib, Malak Amro, Siham Atari, Reema Tayyem, Khlood Bookari, Noor Alawadhi, Sabika Allehdan, Hana Trigui, Elie Sokhn, Yousef Khader, Eman Badran, Iman Kamel, Atiyeh Abdallah, Mohamed Jemaà, Emmanuel Musa, Jude Dzevela Kong

**Affiliations:** ^1^ Department of Computer Science, Al-Quds University, Jerusalem, Palestine; ^2^ Department of Computer Engineering, Istinye University, Istanbul, Türkiye; ^3^ Department of Public Health, College of Health Sciences, Abu Dhabi University, Dubai, United Arab Emirates; ^4^ Department of Human Nutrition, College of Health Sciences, Qatar University, Doha, Qatar; ^5^ Department of Clinical Nutrition, Faculty of Applied Medical Sciences, Taibah University, Medina, Saudi Arabia; ^6^ School of Education, Kuwait University, Kuwait City, Kuwait; ^7^ Department of Biology, College of Science, University of Bahrain, Zallaq, Bahrain; ^8^ Laboratoire de Microbiologie Moléculaire, Vaccinologie et Développement Biotechnologique, Institut Pasteur de Tunis, The University of Tunis El Manar, Tunis, Tunisia; ^9^ Molecular Testing Laboratory, Medical Laboratory Department, Faculty of Health Sciences, Beirut Arab University, Beirut, Lebanon; ^10^ Department of Public Health, Faculty of Medicine, Jordan University of Science and Technology, Irbid, Jordan; ^11^ Neonatal-Perinatal Division, Pediatric Department, Faculty of Medicine, University of Jordan, Amman, Jordan; ^12^ National Research Centre, Cairo, Egypt; ^13^ Department of Biomedical Sciences, College of Health Sciences, QU Health Sector, Qatar University, Doha, Qatar; ^14^ Human Genetics Laboratory, Faculty of Medicine of Tunis, Tunis El Manar University, Tunis, Tunisia; ^15^ Neurophysiology, Cellular Physiopathology and Valorisation of Biomolecules Laboratory, Faculty of Sciences of Tunis, Tunis El Manar University, Tunis, Tunisia; ^16^ Department of Biology, Faculty of Sciences of Tunis, Tunis El Manar University, Tunis, Tunisia; ^17^ AI4PEP, York University, Toronto, ON, Canada; ^18^ Department of Mathematics and Statistics, Faculty of Science, York University, Toronto, ON, Canada

**Keywords:** digital health literacy, health management, digital determinants of health, machine learning, Arab countries, MENA region

## Abstract

**Background:**

Digital health literacy is a critical digital determinant of health (DDoH) in the Arab Middle East and North Africa (MENA) region, where technological disparities, limited healthcare infrastructure, and diverse socio-cultural contexts significantly impact healthcare access and management.

**Objective:**

This study evaluates the impact of digital health literacy on health management practices and ensuing health outcomes in Arab Countries, employing predictive modeling as an analysis tool to uncover key determinants.

**Methods:**

A cross-sectional survey of 12,522 respondents from ten Arab MENA countries was analyzed to examine relationships between survey features and health outcomes. We compared multinomial regression to machine learning models, including CatBoost and Random Forest, to predict outcomes and identify significant predictors.

**Results:**

CatBoost, a powerful ML model that handles categorical data efficiently, achieved a predictive accuracy of 97.8%, outperforming other models in capturing complex, nonlinear relationships. Five key determinants of digital health literacy on health management outcomes were identified: limited internet access, restricted health service access, confidence in AI health resources, health monitoring tool usage, and social media health information consumption.

**Conclusion:**

Enhancing digital health literacy is critical for improving healthcare outcomes in the Arab MENA region. This study underscores the need for culturally tailored digital health interventions to address regional technological and healthcare challenges. Policymakers must prioritize these strategies to reduce disparities and empower individuals in managing their health.

## 1 Introduction

Digital Determinants of Health (DDoH) refer to the technological factors within digital environments that influence an individual's health status and healthcare experience by enhancing access to affordable, quality healthcare services (1, 2). Key DDoH include, but are not limited to, access to technological tools, digital literacy, digital accessibility, digital availability, digital affordability, technology personalization, data poverty, information asymmetry, and internet connectivity (1, 3).

One key Digital Determinants of Health (DDoH) is digital accessibility, which refers to individuals' access to the internet and necessary devices, and digital affordability, which addresses the financial burden associated with digital services and hardware. Another important factor is data poverty, a condition in which people lack adequate access to digital data, limiting their ability to make informed decisions. Additionally, digital literacy plays a crucial role, as it encompasses the skills required to use digital tools effectively and safely (3).

Studies suggest that DDoH, particularly digital health literacy, are positively associated with improved health outcomes. Individuals with higher digital health literacy are better equipped to participate in their medical decisions and demonstrate better psychological states and overall quality of life (4, 5). Similarly, internet usage, as a form of digital health literacy, is independently associated with certain health behaviors such as exercise and balanced nutrition (6).

DDoH are a subset of, and often interact with, social determinants of health (SDoH), which encompass the circumstances in which people are born, live, learn, work, play, worship, and age. Together, these factors are responsible for health inequities (2, 7). Disparities in digital access and literacy can exacerbate existing health inequalities. The concept of the digital divide posits that individuals with limited access to digital technologies or low digital literacy levels face disadvantages in accessing health information and services, thereby worsening health outcomes.

In response to these challenges, digital inclusion has emerged as a necessary approach to close this gap. Defined by the Pan-American Health Organization as “appropriate access, digital skills, and usability and navigability in the development of technological solutions”, digital inclusion seeks to counteract the digital divide by ensuring equal access to technology (8). Without digital inclusion, the digital divide continues to aggravate social and economic barriers, creating further obstacles to healthcare delivery and overall well-being, thus making it a critical SDoH.

The digital divide is especially relevant in Arab Middle Eastern and North African (MENA) countries, where significant disparities in digital access exist among various socioeconomic and demographic groups (9). Despite high literacy rates and a robust digital infrastructure, digital inequalities persist due to the region's heterogeneous cultural, economic, and historical legacies. For example, social inequalities between the wealthy Gulf Cooperation Council (GCC) countries and less-developed areas in the Levant and North Africa are evident in the digital realm. Addressing these disparities is crucial to ensure equitable access to healthcare and foster a more inclusive digital society in the Arab MENA region (10).

In addition to infrastructure and access, cultural factors play a significant role in the relationship between digital determinants and health in the Arab MENA region. Studies have shown that cultural beliefs can influence the use of digital health tools, with cultural attitudes towards technology and healthcare either facilitating or hindering the adoption of digital interventions. In conservative societies, for example, resistance to using digital tools for health-related purposes, particularly among older populations, may arise. A review of digital health in fragile states within the MENA region underscores persistent barriers to implementation, including low levels of computer literacy, underdeveloped technological infrastructure, and concerns regarding data privacy. These challenges significantly hinder the integration of digital health technologies and contribute to the deepening of existing health disparities (11).

Social media usage affects digital health literacy and plays a key role as a DDoH by influencing health behaviors and spreading health information (12). However, despite high social media usage, trust in these platforms is generally lower in the MENA region compared to other parts of the world (13). Social media platforms shape health perceptions and behaviors, especially among younger populations. While they can serve as valuable sources of health information, they also facilitate the spread of misinformation, which negatively impacts health outcomes. Younger individuals, although adept at using technology, are particularly vulnerable to misinformation, leading to unhealthy behaviors (14). In parts of the Arab MENA region with lower media literacy or unequal access to verified health sources, social media can skew health perceptions and influence behaviors adversely (13).

While some literature exists on digital determinants in the MENA region, there is limited understanding of how these factors specifically influence health outcomes within this context. This gap is particularly significant given that lower levels of digital literacy in certain areas may lead to disparities when compared to findings from other regions. Moreover, traditional statistical methods often fall short in capturing the complex, non-linear relationships among multiple digital factors across the diverse populations of the Arab MENA region.

Machine learning (ML) has emerged as a powerful tool for analyzing large-scale health datasets to extract population-level healthcare outcomes (15). The advent of artificial intelligence and ML has become instrumental in delivering personalized healthcare with improved quality, speed, and precision. Over the years, ML has been used to assist with case triage and diagnoses, enhance image scanning and segmentation, support decision-making, predict disease risk, and contribute to neuroimaging, among other applications (16–21). These advancements enable the prediction of health emergencies and disease populations, highlighting AI's growing role in healthcare (22).

Despite its potential, applying ML to study the impact of digital determinants on health is still in its early stages. For example, ML has been utilized to predict and detect mental illness through social media, identifying specific behavioral patterns (23, 24). These studies highlight ML's ability to uncover nuanced relationships between digital determinants and health outcomes that may not be apparent through traditional statistical methods.

In Arab MENA countries, the application of ML to analyze the impact of digital determinants on health remains underexplored. However, several studies have begun to examine this intersection, showcasing ML's ability to provide deeper insights into how digital factors influence health in this region. For example, researchers applied ML techniques to analyze health data in Saudi Arabia by developing a big data analytics system for healthcare (25). This system analyzed data content on social media platforms - Twitter in this case - demonstrating that ML could effectively extract valuable healthcare data from social digital determinants such as social media usage, offering useful insights for healthcare providers and policymakers.

ML has also been explored for telemedicine solutions in Arabic-speaking countries. Habib et al. found that a predictive text system for medical recommendations in telemedicine models could enhance doctor-patient interactions, save time, and improve service satisfaction (26). This could indirectly influence health outcomes by optimizing medical recommendations. Accordingly, this study investigates the potential of ML models to deepen our understanding of how digital determinants influence health outcomes in Arab MENA countries, particularly where traditional health data may be limited or fragmented.

The relationship between digital determinants and health outcomes is a growing area of research, particularly relevant to the Arab MENA region. Key digital factors such as internet access, and social media usage influence digital literacy and are increasingly recognized for their influence on health outcomes. This study builds on the emerging field of digital health by applying machine learning techniques to analyze relevant data collected through a survey conducted as part of this study. By leveraging the predictive capabilities of machine learning, we aim to understand the interaction among digital health literacy determinants and their influence on health management practices and ensuing health outcomes, ultimately contributing to more targeted and effective public health strategies that address the unique public health challenges in Arab MENA countries. As such, this study is among the first to apply machine learning to assess digital health literacy in the Arab MENA region at scale.

## 2 Materials and methods

### 2.1 Study design and data collection

The research approach included a cross-sectional survey to examine the digital determinants of health in 10 Arab countries within the Middle East and North Africa (MENA) region: Bahrain, Palestine, Lebanon, Jordan, Kuwait, the UAE, Saudi Arabia, Egypt, Morocco, and Tunisia. The study aimed to explore the impact of digital literacy on health management practices and health outcomes through self-reported assessments of the impact thereof (*perceived* impact). Data were collected through a structured online questionnaire that was made available online through the Al-Quds University platform in both Arabic and English to accommodate language preferences.

In this study, data collection was conducted through a self-administered online questionnaire distributed via the Al-Quds University platform, meaning there were **no physical data collectors**; instead, dissemination relied on **digital outreach channels** including social media, university networks, and community forums across the 10 participating Arab countries. The **validity of the questionnaire** was ensured through a two-step process: first, it was reviewed by **country-based expert partners** to establish face validity, and second, it underwent a **pilot test** among a sample from the target population to refine clarity and relevance. Data collection ran from June of 2024 to August of the same year, and the survey involved 12,522 respondents. Moreover, to ensure representation across the 10 countries, we aimed for a minimum of 200–300 responses per country. Moreover, A minimum of 200–300 participants per country was targeted to ensure statistically reliable estimates, meaningful cross-country comparisons, and sufficient power for subgroup analyses.

We conducted this study in accordance with the ethical principles outlined in the Declaration of Helsinki. Ethical approval was obtained from Institutional Review Boards (IRBs) of participating countries (Approval Date: March 30, 2024; Ref No: 384/REC/2024). All participants provided informed consent prior to inclusion in the study.

### 2.2 Data preprocessing

To ensure data quality and representational balance, we inserted the dataset into a rigorous preprocessing pipeline where we prepared the data for analysis. Our pipeline comprised the following steps: (1) encoding categorical variables. In this step we converted all categorical data into numerical format to standardize it. (2) Handling missing values using mode imputation (27), which was an appropriate choice as our dataset consisted exclusively of categorical variables. Mode imputation is particularly well-suited for categorical data as it preserves the distribution of categorical values by replacing missing entries with the most frequently occurring category. (3) To address potential sampling bias and ensure representative dataset characteristics across gender and age, we utilized the Minority Over-sampling Technique (SMOTE) combined with Edited Nearest Neighbors (ENN) algorithm (28, 29). This last step also helped eliminate potentially noisy or outlier data points, thereby improving the dataset's overall quality and predictive reliability. This sampling method is particularly powerful as it minimizes potential sampling biases while preserving the original data's fundamental characteristics and size. To ensure transparency, all these steps, including transformations, groupings, and balancing, were directly applied to the original survey provided.

### 2.3 Study features

The study aimed to provide a nuanced understanding of how digital health literacy influences health management outcomes in the Arab MENA region. To achieve this, we designed the survey to capture the multifaceted dimensions of this impact. Table 1 outlines the features collected by the survey, including internet access and usage, online health-seeking behaviors, health information sources, online health community engagement, and participants' confidence in and understanding of online health information. Additionally, demographic data were collected to contextualize the target variable - namely, the overall impact of digital literacy on health management - across diverse populations in Arab MENA countries. These variables collectively served as predictors for the target.

**Table 1:** Predictive determinants of digital health management across the Arab Mena region.

Category	Features
Target variable	The overall impact of digital literacy on my health management
Sociodemographic Information	Age group, Sex; Country of Residence; Area of Residence, Level of Education, Employment Status
Internet access and usage	Internet Connection Consistency; Lack of internet access limits my ability to access health services; Using the internet to make a decision on how to treat an illness or health condition; Confident in using search engines; Use of social media to access or share health information
Health information seeking behavior	Lack of Access limited the ability to access health services; Ability to distinguish between reliable and unreliable health information; Searching for general health information; Looking up symptoms or conditions; Booking medical appointments online; Health Monitoring Services
Health information sources	Government health websites; Hospital or clinical websites; Health-focused news sites; Universities and Educational Institutions websites; Health forums and communities; Social media platforms; Blogs and personal websites
Online health community engagement	Participation in online forums or communities for health support; following health and wellness influencers or pages; Participating in health-related groups or forums
Health information confidence and understanding	Confident in using health information online sources; Confident in using health Apps; Confident in understanding and using online health information for decision making; Confident in using ChatGPT and AI Apps

### 2.4 Statistical analysis

We first performed descriptive analytic tests to summarize and characterize the dataset and get an official overview of the research population. This step aimed to examine the relationships between the predictor variables and the target. Factors including country of residence, age, education level, internet access, sources of health information, and other digital health literacy indicators were analyzed using frequency distributions and percentages. Moreover, we implemented the multinomial logistic regression classification method to explore the associations between the variables in the feature set and health management outcomes.

### 2.5 Machine learning analysis

Whereas traditional statistics derives population inferences from a sample, machine learning (ML) can identify generalizable predictable patterns. Accordingly, this study utilized multiple ML models to predict the impact of digital health literacy on health management outcomes in the region.

#### 2.5.1 Decomposing the classification problem

Our research problem was originally a 3-class classification task, where the target variable had three possible responses: positive, neutral, or negative. For this study, we decomposed the original multi-class classification problem into two distinct binary classification tasks. Here the “neutral” class served as the reference or control group, allowing us to study the “positive” and “negative” impacts separately. This approach simplified the problem, enhanced conceptual clarity, and reduced algorithmic and computational complexity.

This binary decomposition was also chosen to improve model interpretability and facilitate targeted analysis. By isolating the positive and negative impacts relative to a neutral baseline, we were able to derive more actionable insights into the distinct drivers of beneficial vs. adverse digital health literacy outcomes. A multinomial approach, while feasible, would have introduced additional complexity and made it more difficult to disentangle these opposing effects in a meaningful and interpretable way.

The resulting predictive modeling schemes, neutral-negative and neutral-positive, focused on analyzing variables with a negative and positive impact on the outcome variable, respectively.

#### 2.5.2 Algorithms and techniques

We applied and compared the performance of multiple ML models to comprehensively evaluate the impact of digital health literacy on health management outcomes. The selection of algorithms was guided by three primary criteria: (1) proven effectiveness for categorical data classification tasks, (2) diverse algorithmic approaches to capture various data patterns, and (3) model interpretability to derive actionable insights from the analysis. All preprocessing and machine learning implementations were conducted using Python 3.9, while statistical analyses were performed using IBM SPSS Statistics v27.

Algorithms and models we used included support vector machines (SVM), random forest (RF), CatBoost, gradient boosting (GB), and decision trees (DT). SVMs are excellent at detecting complex, non-linear correlations by generating optimal hyperplanes that split distinct data classes (30). Random forest, an ensemble learning technique, builds several decision trees to make more accurate predictions and manage a wide range of variable types (31). CatBoost, a gradient boosting toolkit, excels in processing categorical features effectively, reducing overfitting (32). Gradient boosting combines multiple weak learners sequentially, with each new model focusing on correcting previous models' errors, thereby progressively improving predictive accuracy (33). Decision trees provide interpretable models by recursively splitting datasets based on attribute decisions, making them valuable for understanding complex health-related interactions (34).

This diverse set of algorithms, where each contributed unique strengths to the analysis, enabled a more thorough investigation of the complex interactions between digital health literacy and management practices, resulting in strong and insightful results.

#### 2.5.3 Hyperparameter optimization

The models described in Section 2.5.2 each have a set of configuration variables internal to them called hyperparameters. Unlike model parameters that are learned from data during training (such as weights in neural networks or coefficients in regression models), hyperparameters are external configuration settings that control the learning process itself and must be set before training begins. Fine-tuning these hyperparameters enables researchers to enhance a model's predictive accuracy and ability to generalize to unseen data by balancing complexity and overfitting. The process of hyperparameter optimization involves systematically adjusting and evaluating combinations of hyperparameters to achieve optimal performance metrics. For this study, we employed grid search with 5-fold cross-validation using accuracy as the primary optimization metric. Prior to hyperparameter tuning, we established baseline performance using default configurations for each algorithm to identify which models warranted further optimization.

Table 2 provides a detailed summary of the hyperparameters explored for each machine learning model we used. The table outlines the key hyperparameters, their assigned values, and corresponding descriptions.

**Table 2:** Hyperparameter optimization of machine learning models.

ML models	Hyperparameters	Value
RF	Number of decision trees (n_estimators)	100
	Maximum depth of trees (max_depth)	10
	Number of features to consider for each split (max_features)	“auto”
SVM	Kernel type	“rbf”
	Regularization parameter (C)	1.0
	Kernel-specific parameters (*γ*)	“scale”
GB	Learning rate (*η*)	0.1
	Number of boosting stages (n_estimators)	100
	Maximum depth of individual trees (max_depth)	3
	Subsample fraction (subsample)	1.0
DT	Maximum depth of the tree (max_depth)	10
	Minimum samples required to split (min_samples_split)	2
	Minimum samples required at a leaf node (min_samples_leaf)	1
CatBoost	Learning_rate	0.01
	l2_leaf_reg	1
	Iterations	1,000
	Depth	8
	Border_count	128

### 2.6 Validation process

To validate the findings of our ML models and ensure their reliability, we implemented 10-fold cross-validation. Following this method, we divided the dataset into 10 equal parts (folds). Each fold served as the test set exactly once, while the remaining 9 folds were used as the training set. This process was repeated 10 times, with each fold serving as the test set in a different iteration. By rotating through every data point, 10-fold cross-validation minimizes bias and provides more reliable and generalizable estimates of model performance. To further mitigate the risk of overfitting, we conducted an additional hold-out validation. We reserved 15% of the dataset as an unseen test set, which was not used during training or cross-validation. Performance on this external set remained consistent with cross-validation results, supporting the generalizability of the model.

To assess the models' predictive reliability, we measured multiple performance metrics during each iteration of the cross-validation process. These metrics included accuracy, precision, recall (sensitivity), F1-score, Matthews’ correlation coefficient (MCC), and the Area Under the Receiver Operating Characteristic Curve (AUC) (35, 36). Higher metric values indicate better model performance. Ultimately, we averaged these values across the 10 folds to provide a comprehensive assessment of performance. Moreover, to identify the most influential factors affecting health management, we used CatBoost to determine the top 20 most important features. Additionally, we plotted SHAP (SHapley Additive exPlanations) values to visually interpret the contribution of each feature to the models' predictions (37). By combining these feature analysis techniques with robust validation, this research provided a reliable evaluation of performance and actionable insights into the role of digital literacy in health management.

## 3 Results

### 3.1 Descriptive analysis

Table 3 presents the distribution of impact levels of digital health literacy across the various demographic groups examined in this study. This table illustrates how digital health literacy influences health habits and decision-making across the diverse Arab MENA region.

**Table 3:** Distribution of impact levels of digital literacy on healthy behaviors and decision-making among different demographic groups in the Arab MENA region.

Features	Negative *n* (%)	Neutral *n* (%)	Positive *n* (%)
Age group			
18–34	1,051 (37)	1,485 (32.9)	1,638 (31.7)
35–44	1,115 (39.2)	1,586 (35.2)	1,473 (28.5)
45–64	677 (23.8)	1,437 (31.9)	2,060 (39.8)
Sex			
Male	1,417 (49.8)	1,948 (43.2)	2,896 (56)
Female	1,426 (50.2)	2,560 (56.8)	2,275 (44)
Area of residence			
Urban	2,307 (81.1)	4,015 (89.1)	4,622 (89.4)
Non-Urban	536 (18.9)	493 (10.9)	549 (10.6)
Country			
Bahrain	78 (22.4)	223 (64.1)	47 (13.5)
Egypt	79 (5.3)	440 (29.6)	969 (65.1)
Jordan	255 (11.6)	1,099 (50)	842 (38.3)
Kuwait	346 (25.3)	716 (52.3)	306 (22.4)
Lebanon	146 (16.3)	440 (49.2)	308 (34.5)
Morocco	334 (25.5)	591 (45.2)	383 (29.3)
Palestine	147 (12.3)	479 (40.1)	568 (47.6)
Saudi Arabia	288 (15.3)	933 (49.5)	663 (35.2)
Tunisia	65 (10.4)	271 (43.4)	288 (46.2)
United Arab Emirates	59 (4.8)	519 (42.6)	640 (52.5)
Educational level			
≤Secondary	688 (24.2)	787 (17.5)	534 (10.3)
Bachelor	1,271 (44.7)	2,124 (47.1)	2,509 (48.5)
Graduate	884 (31.1)	1,597 (35.4)	2,128 (41.2)
Employment status			
Employed	1,412 (49.7)	2,787 (61.8)	3,327 (64.3)
Unemployed	785 (27.6)	955 (21.2)	1,258 (24.3)
Student	646 (22.7)	766 (17)	586 (11.3)
Internet consistency			
Unreliable	514 (18.1)	493 (10.9)	427 (8.3)
Moderate reliable	1,008 (35.5)	1,956 (43.4)	1,639 (31.7)
Reliable	1,321 (46.5)	2,059 (45.7)	3,105 (60)

The findings in Table 3 highlight how different groups across the Arab MENA region experience the impact of digital literacy on their health behaviors and decisions in varied ways. Age seems to make a difference—while younger adults (18–34) tended to report more negative or neutral effects, older adults (especially those aged 45–64) were more likely to say that digital tools had a positive impact on their health management. When it comes to gender, men were more likely than women to feel that digital literacy helped them make better health decisions. In contrast, women more often reported neutral experiences, which might reflect differences in how men and women use or access digital health information.

People living in urban areas reported positive or neutral impacts, while those in non-urban areas were underrepresented across the board. This pattern points to the ongoing digital divide between urban and rural communities, where access and infrastructure may still be uneven.

Looking at the results by country, some clear contrasts emerge. In Egypt, the UAE, and Tunisia, a majority of respondents felt that digital tools had a positive impact on their health. On the other hand, in Bahrain and Kuwait, more people reported either no effect or even a negative impact. These differences might reflect local differences in digital health services, infrastructure, or even cultural factors.

Education also played a clear role. The more educated the respondents, the more likely they were to say digital literacy helped them. Those with only a secondary education or less were far less likely to report positive outcomes, reinforcing the idea that digital health literacy is closely tied to general education levels. Furthermore, employment status also mattered. Employed people were more likely to see positive results from using digital tools, while students were the least likely to report a benefit. This could be because working adults might use digital tools more for practical health management. Finally, reliable internet access was crucial. Among those with good connectivity, a strong majority said digital literacy helped them. But for those with unreliable internet, the positive impact was much lower.

### 3.2 Negative impact analysis

Table 4 compares feature importance using three ranking methods: CatBoost's SHAP values, Random Forest importance, and multinomial logistic regression (odds ratios and *p*-values). These methods highlight the significance of features in explaining the negative impact of digital health literacy on health management outcomes.

**Table 4:** Comparison of feature importance across models for negative impact of digital literacy on health management.

Rank^a^	Feature	RF Importance (%)	Odds Ratio [Exp (B)]	*P*-value
1	LackInternetAccess	48.42	0.705	<0.001
2	GovtHealthWebsites	36.71	1.103	0.034
3	CountryResidence	70.99	1.001	0.471^c^
4	HospitalWebsites	31.23	1.379	0.001
5	SocialMediaPlatforms	37.11	1.549	0.001
6	Blogs	30.87	0.891	0.005
7	SocialMediaUsage	33.09	0.694	<0.001
8	HealthNewsSites	34.9	1.379	0.001
9	HealthMonitoring	29.35	1.267	0.003
10	LimitAccessHealth	51.55	0.596	<0.001
11	BookingAppointments	29.22^b^	0.897	0.003
12	EmploymentStatus	30.87	1.098	0.011
13	FollowingInfluencers	30.7	1.003	0.92^c^
14	HealthInfoUnderstanding	29.34	1.038	0.359^c^
15	InternetDecisionMaking	32.71	1.038	0.002
16	UniversityHealthWebsites	30.68	0.899	0.003
17	HealthForums	30.86	1.219	<0.001
18	HealthAppsConfidence	32.81	1.122	<0.001
19	AIAppsConfidence	33.19	1.267	<0.001
20	AgeGroup	34.9	0.876	0.001

^a^ According to CatBoost's SHAP values.

^b^ Out of top 20 features by RF.

^c^ Failed to detect the significance.

CatBoost excels at identifying the most relevant predictors by handling complex interactions between features. It uses gradient boosting, a method where multiple trees are built sequentially, each correcting the errors of the previous one. In contrast, Random Forest also captures non-linear relationships but ranks more features as important due to its random feature selection at each split, which can sometimes result in less relevant features receiving higher importance scores. Multinomial regression, on the other hand, focuses on linear relationships and highlights features based on statistical significance. However, it does not account for interactions between variables, which can cause it to overemphasize certain features.

According to Random Forest rankings, *Country of Residence* is the most significant feature with an importance score of 70.99%, followed by *Limited Access to Health Services* at 51.55% and *Lack of Internet Access* at 48.42%. Moreover, the model assigns considerable importance to features such as *Using Government Health Websites* (36.71%) and *Employment Status* (30.71%), which are ranked lower in other models. This may stem from Random Forest's sensitivity to non-linearities, potentially inflating the importance of less relevant features (REF). However, Random Forest's inclusion of a larger number of variables can introduce noise, potentially diluting its ability to precisely identify the key drivers of health management outcomes.

**Figure 1:**
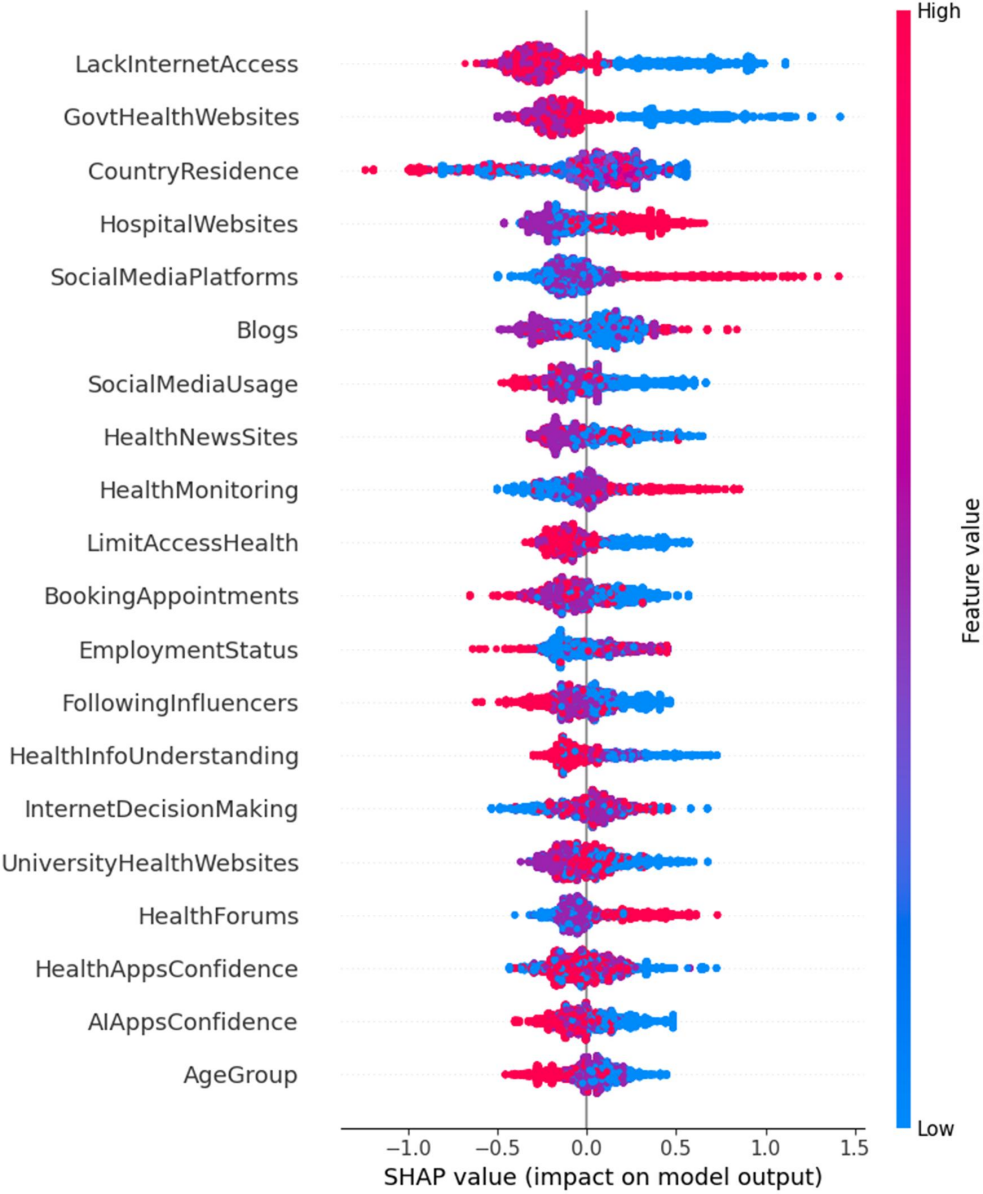
Feature importance for negative impact on digital health literacy and management.

The CatBoost model, as illustrated in Figure 1, offers a slightly nuanced ranking of feature importance, identifying *Limited Access to Health Services*, *Lack of Internet Access*, and *Confidence in AI Apps* as the primary contributors to the negative impact of digital literacy on health outcomes. Notably, *Confidence in AI Apps,* which ranked lower in random forest, is a highly significant predictor according to CatBoost. This is because the strength of CatBoost lies in its ability to detect intricate interactions among variables, ensuring that only the most relevant features are prioritized while minimizing the influence of less impactful ones. This approach provides a clearer and more precise understanding of the factors influencing health outcomes, particularly in the context of this study.

Moreover, CatBoost's enhanced interpretability through SHAP values further enhances its utility. For example, *Limited Access to Health Services* and *Lack of Internet Access* are shown to have strong negative impacts on health management, whereas *Confidence in AI Apps* emerges as a significant driver of positive outcomes. This level of interpretability is a noteworthy advantage of CatBoost, as it offers deeper insights into how each feature influences the overall prediction process.

The regression analysis helps us understand which factors influence whether people feel digital tools help or hinder their ability to manage their health. In this context, the beta coefficients [reported as Exp(B)] show the strength and direction of each factor's impact. When Exp(B) **is** less than 1, it means the factor is associated with a *lower* likelihood of effective health management. For example, people who reported limited access to health services were 41% less likely to benefit from digital health tools, while those with unstable internet access were about 30% less likely to manage their health effectively. These results aren't surprising—without basic access, digital tools simply can't function as intended.

On the other hand, positive beta values [Exp(B) greater than 1] reflect a *higher* likelihood of improved health management. People who had confidence in AI-powered health apps, or who regularly used official hospital or health news websites, were significantly more likely to say that digital tools helped them make better health decisions. For instance, confidence in AI apps increased the likelihood of reporting a positive impact by 26.7%.

The regression model did not detect the significance of three following features: *Country of Residence*, *Following Influencers*, and *Understanding Health Info*, which had *P*-Values of 0.471, 0.92, and 0.359, respectively. Moreover, and while *Education Level* appeared significant in both the multinomial regression and Random Forest models, CatBoost did not recognize its impact as a predictor of negative health management outcomes. This discrepancy is likely due to CatBoost's ability to identify complex correlations and dependencies between variables. In digital health management, factors like Understanding Health Info, Health Monitoring, and Using Health News Sites may have a more significant negative impact than Education Level, despite their indirect correlation. Furthermore, the diminished importance of *Education Level* in the CatBoost model suggests that formal education alone may not be as critical to negatively impacting digital health management as previously assumed. Instead, specific health-related knowledge and the capacity to understand and apply health information may play a more significant role. This aligns with Norman's concept of eHealth literacy, which encompasses a broader set of skills beyond general literacy, such as the ability to understand and apply health information (38). Interestingly, even though CatBoost and Random Forest had nearly identical accuracy scores (0.9728 vs. 0.9718), declaring CatBoost the “best” based on that tiny difference might be misleading. What really sets CatBoost apart is its ability to detect hidden relationships between features—something traditional regression can't easily do.

### 3.3 Positive impact analysis

Table 5 compares the significance of features in explaining the positive impact of digital health literacy on health management outcomes. Random Forest (RF) highlights the importance of demographic features, ranking *Age Group* (78.61%) and *Sex* (46.07%) as the most significant predictors. Additionally, RF includes a range of broader features like *Searching for General Health Info* and *Country of Residence,* which reflect general patterns but lack the specificity of the targeted behaviors captured by CatBoost.

**Table 5:** Comparison of feature importance across models for positive impact of digital literacy on health management.

Rank^a^	Feature	RF importance (%)	Odds ratio [Exp(B)]	*P*-value
1	HealthMonitoring	31.23	1.22	0.001
2	CountryResidence	37.49	0.99	0.471^c^
3	InternetConnection	33.74	0.99	0.05
4	AgeGroup	78.61	1.15	<0.001
5	FollowingInfluencers	32.58	1.13	0.03
6	Sex	46.07	0.57	<0.001
7	SearchEngine	32.37	1.05	0.001
8	LookupSymptoms	27.94^b^	1.293	<0.001
9	DistinguishInformation	32.73	1.03	0.005
10	EducationLevel	34.16	1.33	0.001
11	SocialMediaPlatforms	33.09	0.69	0.001
12	InternetDecisionMaking	32.71	1.04	0.002
13	HealthNewsSites	34.9	1.38	0.001
14	LimitAccessHealth	32.78	0.85	0.004
15	HealthForums	30.86	1.22	0.001
16	HealthGroupsParticipation	27.83^b^	0.941	0.131^c^
17	BookingAppointments	29.22	0.89	0.003
18	AIAppsConfidence	30.73	1.1	0.001
19	Blogs	30.87	0.89	0.005
20	HospitalWebsites	31.23	1.37	0.001

^a^ According to CatBoost's SHAP values.

^b^ Out of top 20 features by RF.

^c^ Failed to detect the significance.

**Figure 2:**
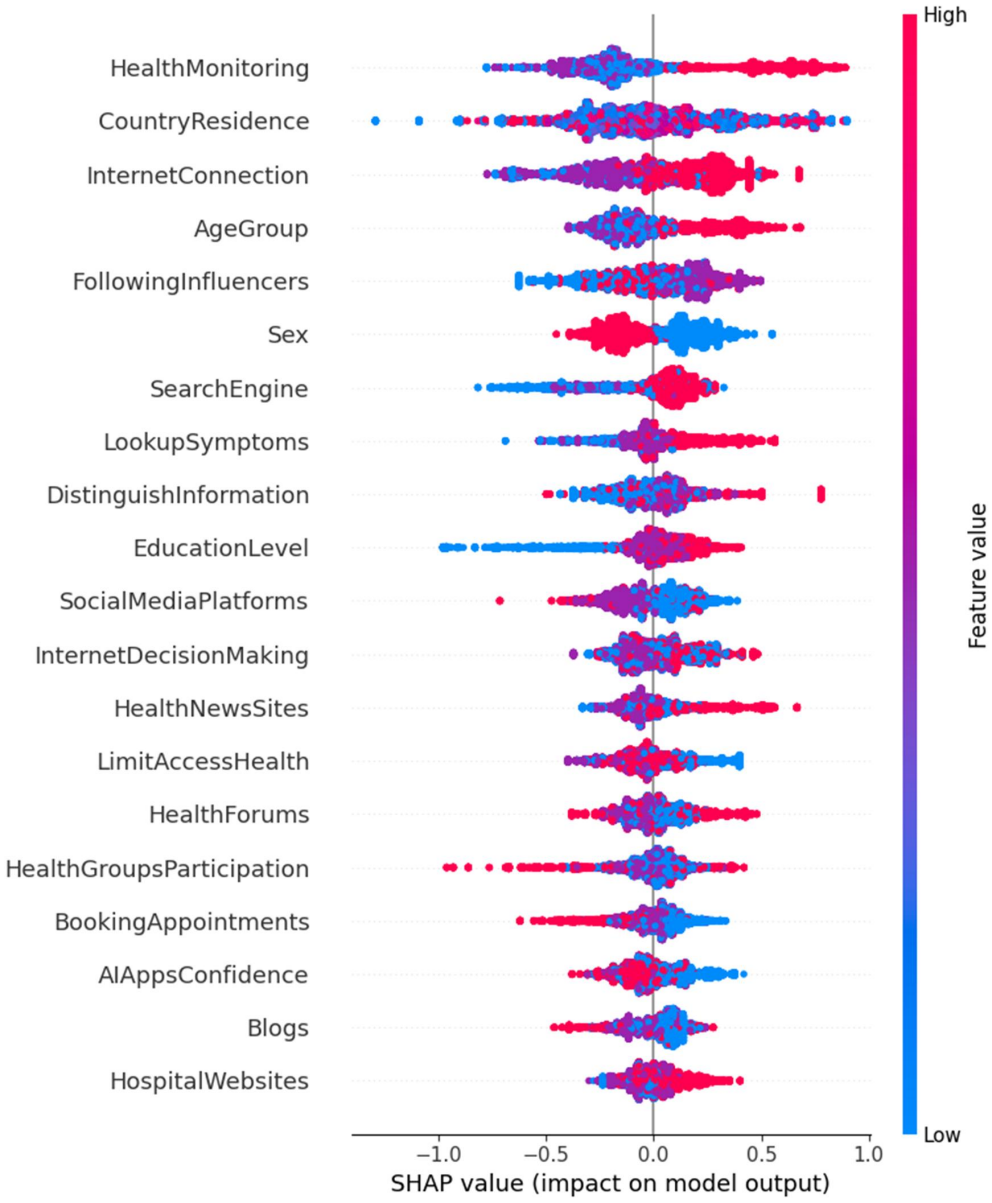
Feature importance for positive impact on digital health literacy and management.

In contrast, CatBoost (Figure 2) emphasizes behavior-driven predictors, offering a more refined perspective on positive health management outcomes. For instance, *Health Monitoring* is the highest-ranked feature in CatBoost, with a significant odds ratio [Exp(B) = 1.22, *p* = 0.001]. This underscores the importance of regular engagement with digital health tools. Interestingly, *Health Monitoring* ranks much lower (18th) in RF, suggesting that CatBoost's non-linear modeling better captures the value of proactive health behaviors.

CatBoost also highlights the significance of features such as *Confidence in AI Apps* [Exp(B) = 1.10, *p* = 0.001] and *Following Influencers* [Exp(B) = 1.13, *p* = 0.03], emphasizing the growing role of trust in AI tools and social influence on health-related decision-making. These insights underscore the shift toward personalized, behavior-driven approaches in health management. Other behavior-specific features, like *Looking up Symptoms* [Exp(B) = 1.29, *p* < 0.001] and *Health Groups Participation* [Exp(B) = 0.94, *p* = 0.131], reinforce the importance of targeted actions in achieving positive health outcomes.

The multinomial regression model (Table 4) offers additional insights by identifying significant linear relationships, such as *Health Monitoring* [Exp(B) = 1.22, *p* = 0.001] and *Search Engine Usage* [Exp(B) = 1.05, *p* = 0.001]. These findings align with CatBoost's emphasis on behavior-specific features. However, the regression model does not capture the importance of variables like *Country of Residence* (*p* = 0.471) and *Health Groups Participation* (*p* = 0.131), which are considered significant in RF and CatBoost's non-linear approaches.

In summary, while Random Forest provides a broad but generalized perspective on factors influencing health management through digital literacy, CatBoost refines this focus by emphasizing behavior-driven predictors such as *Health Monitoring*, *Looking up Symptoms*, and *Health Groups Participation*. The multinomial regression model, while useful for identifying linear relationships, lacks the flexibility to capture the full complexity of these interactions that nonlinear models like CatBoost excel at identifying. This comparison highlights the value of non-linear modeling in understanding the intricate dynamics of digital health literacy and its positive impact on health management.

### 3.4 Classification model performance

Table 6 shows the classification performance of the different models across neutral-negative and neutral-positive classifications, highlighting the strengths of CatBoost and Random Forest (RF) in handling complex data relationships. CatBoost consistently outperforms other models, demonstrating its robustness in both scenarios, as shown by metrics such as accuracy, F1 score, and Matthews correlation coefficient (MCC). These metrics provide a comprehensive evaluation of each model's ability to determine the effect of digital health literacy on health management.

**Table 6:** Classification performance comparison of machine learning models for digital literacy in health management.

Model	Classifier	AUC	Accuracy	F1	Precision	Recall	MCC
Neutral-negative	RF	0.9976	0.9718	0.9717	0.9719	0.9718	0.9405
	SVM	0.9828	0.9500	0.9498	0.9502	0.9500	0.8944
	GB	0.9347	0.8605	0.8580	0.8621	0.8605	0.7034
	DT	0.9629	0.9631	0.9632	0.9634	0.9631	0.9226
	CatBoost	0.9974	0.9728	0.9727	0.9729	0.9728	0.9426
Neutral-positive	RF	0.9987	0.9774	0.9774	0.9775	0.9774	0.9546
	SVM	0.9818	0.9447	0.9447	0.9450	0.9447	0.8892
	GB	0.9083	0.8233	0.8232	0.8237	0.8233	0.6452
	DT	0.9751	0.9747	0.9746	0.9745	0.9747	0.9492
	CatBoost	0.9983	0.9777	0.9776	0.9777	0.9776	0.9552

**Figure 3:**
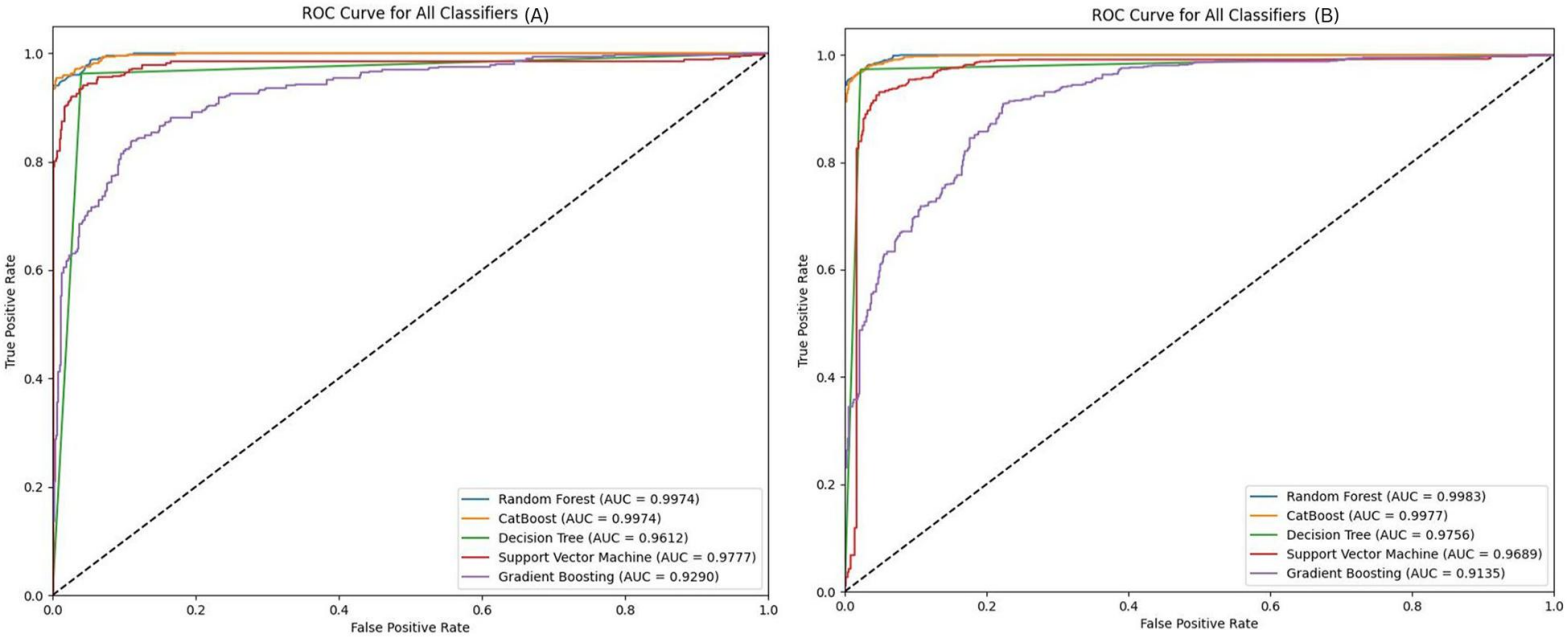
AUC curves comparing different ML models performance in **(A)** neutral-negative and **(B)** neutral-positive classifications.

In the neutral-negative class, as shown in the AUC curve (Figure 3A), CatBoost achieves an accuracy of 0.9728, slightly surpassing RF's 0.9718. This indicates that CatBoost is marginally more effective at correctly classifying positive and negative samples. Both models exhibit excellent performance in terms of precision and recall, but CatBoost edges out RF in terms of the F1 score (0.9727 vs. 0.9717), reflecting its balanced handling of true positives and false positives. CatBoost's AUC of 0.9974 is almost identical to RF's 0.9976, further emphasizing its efficiency in distinguishing between classes. The MCC score, which measures the correlation between true and predicted values, also shows CatBoost's superiority (0.9426) over RF (0.9405), signifying stronger overall performance.

In the neutral-positive classification task, as depicted in the AUC curve (Figure 3B), CatBoost also takes the lead with an accuracy of 0.9777, narrowly outperforming RF's 0.9774. The F1 score for CatBoost (0.9776) reflects its precise handling of both positive and negative cases, ensuring minimal misclassification. CatBoost's AUC of 0.9983 demonstrates its excellent ability to differentiate between the two classes, aligning closely with RF's 0.9987. While RF remains a close competitor in terms of overall performance, CatBoost consistently delivers slightly higher scores, showcasing its overall capacity to model non-linear interactions more effectively.

In addition to this, CatBoost offers better model interpretation through SHAP values, which help explain individual feature contributions. This combination of slightly better performance, improved feature detection (as shown in Tables 4, 5 where CatBoost identified features missed by RF), and enhanced interpretability leads us to consider CatBoost as the best performing model.

Other models, including SVM, Gradient Boosting (GB), and Decision Tree (DT), show lower performance overall, especially in comparison to CatBoost and RF. As shown in Table 6, CatBoost's ability to capture complex patterns within the data allows it to excel, particularly in metrics like MCC and F1 score, which reflect the model's strength in classifying difficult cases. RF remains the most competitive alternative to CatBoost, though it is slightly less effective at capturing the nuances of the data.

## 4 Discussion

This study aimed to explore how digital health literacy (DHL) influences health-related behaviors and decision-making across ten Arab countries in the MENA region. The findings show that while digital health tools have the potential to support healthier lifestyles and informed decision-making, the extent of their impact varies widely depending on individual, social, and regional factors.

A key finding is that age plays an important role in shaping the perceived impact of DHL. Older adults, particularly those aged 45–64, were significantly more likely to report that digital tools had a positive influence on their ability to manage health. This contrasts with younger adults (aged 18–34), who were more likely to report neutral or negative effects. Similar trends have been reported in previous studies, where older individuals—often managing chronic conditions—tend to benefit more from digital health tools due to higher health needs and greater motivation to use such resources (38, 39). Gender differences were also observed. Men were more likely to report a positive impact of DHL, while women more frequently selected neutral responses. This gap may reflect differences in digital confidence, access, and sociocultural expectations. Previous studies suggest that women may face more challenges in navigating digital platforms, especially in contexts where their roles as caregivers limit their available time or resources for digital engagement (40–43). These patterns point to a need for more inclusive digital health solutions that consider both access and usability for different genders.

Place of residence emerged as another significant factor. Urban residents were consistently more likely to report positive or neutral outcomes than those in non-urban areas. This reflects a persistent urban-rural digital divide, where access to high-speed internet and modern health services is more limited outside of urban centers. These findings align with previous work highlighting how infrastructure gaps can restrict the benefits of digital health initiatives for rural populations (44, 45). Country-level differences were also evident. Participants from Egypt, the United Arab Emirates, and Tunisia were among the most likely to report that digital health tools had a positive influence on their health. In contrast, participants in Bahrain, Kuwait, and Morocco were more likely to report neutral or negative experiences. These findings may reflect differences in national eHealth strategies, trust in public digital platforms, or accessibility of online services, as supported by the World Health Organization's regional eHealth reports (46).

Education was a strong predictor of perceived benefit. Respondents with university or postgraduate degrees were far more likely to report positive outcomes compared to those with only secondary education or less. This is consistent with earlier studies that link higher education levels to better digital and health literacy skills (43, 45). However, machine learning models used in this study revealed that formal education may not always be the strongest predictor. Instead, specific behaviors—such as checking symptoms online or trusting AI-powered apps—had a stronger influence on positive outcomes. This supports more recent approaches to defining eHealth literacy, which emphasize functional and applied skills over formal schooling (38, 39, 41).

Employment status also influenced outcomes. Employed individuals were more likely to benefit from digital health tools, possibly due to more frequent use of digital platforms and stronger motivation to manage health efficiently. Students reported the lowest perceived benefits, which may be explained by their generally lower health needs and less frequent interaction with health systems (43, 47).

Internet reliability stood out as one of the most critical factors. Respondents with stable internet connections were far more likely to benefit from digital tools than those with unreliable access. This confirms that infrastructure remains a foundational requirement for effective digital health adoption (48, 49).

To better understand which features, predict negative experiences with digital health, we compared three models: multinomial logistic regression, Random Forest, and CatBoost. All three models identified limited access to health services, unreliable internet, and lack of confidence in AI-powered apps as major barriers to effective health management. Specifically, individuals with limited access to digital health services were 41% less likely to manage their health effectively, while those with poor internet access were 30% less likely—figures that align with findings in other digital health access studies (49–51).

CatBoost provided additional insights by detecting complex patterns and ranking behavioral predictors such as trust in AI and health monitoring higher than demographic characteristics. These findings echo recent research showing that user confidence and engagement with AI applications are increasingly important for positive digital health outcomes (52, 53). In contrast, the regression model failed to detect several features that CatBoost and Random Forest found important, such as participation in health groups or following health influencers, possibly due to its inability to capture non-linear interactions. Interestingly, although education was considered important in the regression and Random Forest models, CatBoost did not rank it highly. This may suggest that digital engagement behaviors matter more than formal education alone. In line with Norman's model of eHealth literacy, which includes critical thinking and problem-solving, these results suggest that individuals with the right digital behaviors can benefit regardless of their formal schooling (38).

When looking at features that support positive outcomes, CatBoost again outperformed traditional models. It identified active behaviors such as health monitoring, looking up symptoms, and trusting AI tools as the most important predictors of positive health management. These behavior-driven predictors were ranked lower by Random Forest, which emphasized broader traits like age and gender. Multinomial regression also captured some of these behaviors (e.g., health monitoring), but missed others, such as group participation and symptom checking.

Despite the small difference in accuracy between CatBoost (97.28%) and Random Forest (97.18%), CatBoost's ability to uncover meaningful relationships and explain individual feature contributions through SHAP values makes it more suitable for understanding the complex interactions that shape digital health experiences. Other research has highlighted the added value of explainable AI in healthcare, especially when performance differences between models are minimal but interpretability is critical (1, 54).

Our study confirms that digital health literacy can help improve health behaviors and decision-making—but only under the right conditions. Infrastructure, access, trust, and personal behaviors all play a major role in shaping outcomes. Countries such as Egypt and the UAE appear to be successfully leveraging digital tools, while others still face barriers that limit their impact. Simply providing internet access or health apps is not enough. To make digital health effective across the MENA region, we need to invest in user-centered design, trust-building, digital education, and inclusive infrastructure that ensures no one is left behind.

## 5 Strengths and limitations

This study fills an important gap by exploring digital health literacy across ten Arab MENA countries—an area often overlooked in global research. While most studies focus on high-income countries, this work highlights how differences in access, infrastructure, and education shape digital health experiences in more diverse settings.

A key strength lies in the use of both traditional statistical methods and machine learning models. This approach allowed us to uncover more complex patterns, such as the importance of confidence in AI apps, which standard models missed. The use of cross-validation further strengthened the reliability of the results across varied populations.

That said, the study has clear limitations. Being cross-sectional, it shows associations—not causality. We cannot say whether digital health literacy leads to better health, or if those who are already managing their health well are more likely to use digital tools. While machine learning helps reveal important patterns, it does not explain cause and effect. These findings should guide future studies that can track changes over time.

Also, the study relies on self-reported data, which may not always be accurate. People might misjudge their digital skills or health behaviors. Objective usage data would improve future research. Lastly, cultural differences across countries likely shape how people engage with digital health, something that deserves deeper exploration through qualitative or localized studies.

## 6 Conclusion

This study underscores the significant role of digital determinants in health management across the Arab MENA region, highlighting both the challenges posed by the digital divide and the potential benefits of enhancing digital health literacy and access to digital health tools. By employing machine learning models like CatBoost and Random Forest, this research identifies critical factors, such as internet access, confidence in digital tools, and social media engagement, that influence health outcomes.

To mitigate regional health disparities, it is essential to prioritize digital inclusion and literacy in public health strategies, focusing on targeted interventions to improve digital skills, particularly in underserved communities, while fostering confidence in the use of digital health tools. Policymakers should develop user-friendly, culturally relevant digital platforms accessible to diverse populations, and future research should explore the long-term impacts of digital literacy and the influence of cultural factors on digital health behaviors, contributing to more effective public health interventions in the Arab MENA region.

Accordingly, we strongly advocate for tailored healthcare initiatives that address gaps in digital health literacy and support the development of infrastructure to meet the evolving demands of a digital world. Ministries of health should (a) prioritize promoting the responsible use of digital tools, especially those powered by artificial intelligence (AI), to improve access to reliable health information; (b) organize community-based digital health literacy workshops to teach individuals how to access, understand, and evaluate online health information; (c) deploy mobile health (mHealth) outreach units to combine access to care with digital training in remote communities; and (d) work with local schools and ministries of education to integrate digital health literacy into school curricula.

## Data Availability

The raw data supporting the conclusions of this article will be made available by the authors, without undue reservation.
